# Elevated Serum Levels of CCL23 Are Associated with Poor Outcome after Resection of Biliary Tract Cancer

**DOI:** 10.1155/2022/6195004

**Published:** 2022-12-01

**Authors:** Christoph Roderburg, Simon Labuhn, Jan Bednarsch, Sven A. Lang, Anne T. Schneider, Linda Hammerich, Mihael Vucur, Tom F. Ulmer, Ulf P. Neumann, Tom Luedde, Sven H. Loosen

**Affiliations:** ^1^Department of Gastroenterology, Hepatology and Infectious Diseases, University Hospital Düsseldorf, Medical Faculty of Heinrich Heine University Düsseldorf, 40225 Düsseldorf, Germany; ^2^Department of Visceral and Transplantation Surgery, University Hospital RWTH Aachen, Pauwelsstrasse 30, 52074 Aachen, Germany; ^3^Department of Hepatology and Gastroenterology, Charité-Universitätsmedizin Berlin, Campus Virchow-Klinikum (CVK) and Campus Charité Mitte (CCM), Augustenburger Platz 1, 13353 Berlin, Germany

## Abstract

**Background:**

Surgical tumor resection is the only potentially curative treatment option for patients with biliary tract cancer (BTC). However, 5-year survival rates are still below 50% mainly due to tumor recurrence. The preoperative identification of ideal surgical candidates has remained a major challenge and easily accessible algorithms including parameters of the individual tumor biology are missing. Chemokine (C-C motif) ligand 23 (CCl23) has been associated with tumor progression in hepatocellular carcinoma (HCC), but its role in the context of BTC is largely unknown. Here, we evaluated circulating levels of CCL23 as potential diagnostic and prognostic biomarker in patients with resectable BTC.

**Methods:**

CCl23 serum levels were analyzed by multiplex immunoassay in a cohort of 119 BTC patients receiving surgical tumor resection as well as 50 healthy control samples and 11 patients with primary sclerosing cholangitis (PSC).

**Results:**

Baseline serum CCL23 levels were significantly elevated in BTC patients compared to PSC patients as well as healthy controls. CCL23 increased the diagnostic sensitivity and specificity of established tumor markers including CA19-9 and correlated with patients' age and makers of systemic inflammation. Elevated preoperative CCL23 levels were associated with a significantly impaired postoperative outcome. BTC patients with a preoperative CCL23 level above the optimal prognostic cut-off value of 702.4 pg/ml showed a median OS of only 110 days compared to 501 days for patients with low initial CCL23 levels. The prognostic value of circulating CCL23 was confirmed in Cox-regression analysis.

**Conclusion:**

Serum levels of CCL23 are elevated in patients with BTC, and high preoperative CCL23 levels were associated with an impaired postoperative survival. CCL23 serum levels could help to identify the ideal surgical candidates for BTC resection in the future.

## 1. Introduction

Biliary tract cancers (BTC) are malignancies originating from the epithelium of the bile ducts [1, 2]. According to their respective anatomy, BTC are subdivided into intrahepatic and extrahepatic tumors. Additionally, gallbladder carcinomas are also classified as BTC [1, 2]. Despite numerous advances in the field of systemic therapy, such as the introduction of molecularly targeted treatments or immunotherapy, the prognosis for many patients with BTC remains poor [1, 3, 4]. Only patients in early tumor stages in which tumor resection is possible to have a chance of long-term cure [5]. However, larger clinical trials have shown that disease recurrence after tumor resection is observed in up to 60% of cases, leading to a 5-years survival rate of less than 50% even for patients who received adjuvant chemotherapy [5]. In addition, extensive surgery is often necessary to allow complete tumor removal. Thus, postoperative complications are common, and it often remains unclear whether or not an individual patient should undergo surgery. Currently, this decision is mostly based on surgical resectability and the patients' performance status, while the tumor biology is not taken into account [6]. Nevertheless, it seems likely that analyses of molecular characteristics of the individual tumor and/or the individual tumor microenvironment may allow stratifying patients into subgroups that will particularly benefit or not benefit from surgical tumor resection [7–10]. Thus, novel, easily accessible biomarkers might help improving the clinical management of patients with BTC.

CCL23 (macrophage-inflammatory protein 3 (MIP-3)) is secreted by different immune cell types including eosinophils, neutrophils, and monocytes [11]. CCL23 acts by activating its receptor CCR1, which has been found on the surface of both cancer cells and cells of the tumor microenvironment (monocytes, macrophages, dendritic cells, lymphocytes, and endothelial cells) [12]. CCL23 induces tumor cell proliferation, stimulates angiogenesis, and acts as a chemoattractant immune effector cells [13, 14]. While a role for CCL23 has been described in various cancers [15, 16], only very limited data on a potential function in the context of BTC exist. In this observational cohort study, we measured serum concentrations of CCL23 in a large cohort of BTC patients undergoing tumor resection between 2011 and 2017 and examined the role of CCL23 as a diagnostic and/or prognostic marker in these patients.

## 2. Patients and Methods

### 2.1. Patient Characteristics and Study Design

This observational cohort study was performed to investigate a potential diagnostic and prognostic role of circulating CCL23 levels in patients undergoing BTC resection. In total, *n* = 119 patients with BTC who received BTC tumor resection at the Department of Visceral and Transplantation Surgery at University Hospital RWTH Aachen were enrolled between 2011 and 2017 (patient characteristics are summarized in Table 1 and Supplementary Table 1). Whole blood samples were taken before surgery and 6-7 days after BTC resection and centrifuged for 10 min at 2000 g. Serum samples were then stored at -80°C until use. BTC was histologically confirmed in the resected tumor sample. We analyzed a total of *n* = 50 healthy and cancer-free blood donors as well as *n* = 11 patients with primary sclerosing cholangitis (PSC) without signs of malignant transformation as a control population. The study protocol was approved by the ethics committee of the University Hospital RWTH Aachen, Germany (EK 206/09) and conducted in accordance with the ethical standards laid down in the Declaration of Helsinki. Written informed consent was obtained from all patients.

### 2.2. Measurement of CCL23 Serum Levels

Serum levels of CCL23 and IL-4 were measured by multiplex immunoassay according to the manufacture's instruction using a Bio-Plex 200 system and Bio-Plex Manager 6.0 software (Bio-Plex Pro Human Chemokine Panel, #171AK99MR2, Bio Rad, Hercules, CA, USA).

### 2.3. Statistical Analysis

Shapiro-Wilk-Test was used to test for normal distribution. Nonparametric data were compared using the Mann–Whitney-*U*-Test or the Kruskal-Wallis-Test for multiple group comparisons. Mann–Whitney-*U*-Test was performed for ad hoc subgroup comparisons between two groups in case of a significant Kruskal-Wallis-Test. Related samples (CCL23 before and after surgery) were compared by Wilcoxon signed-rank test. Correlation analyses were performed using the Spearman's correlation coefficient. Optimal cut-off values for ROC curves were calculated using the Youden-Index method (YI = sensitivity + specificity − 1). Kaplan-Meier curves show the impact of a specific parameter on the overall survival (OS). Log-rank test was performed to test for differences between groups. The optimal prognostic cut-off value was determined by fitting Cox proportional hazard models to the dichotomized survival status and the survival time and defining the optimal cut-off as the point with the most significant split in the log-rank test. The prognostic relevance of variables was also tested in univariate Cox-regression analyses. The hazard ratio (HR) and the 95% confidence interval are displayed. All statistical analyses were performed with SPSS 23 (SPSS, Chicago, IL, USA) and RStudio 1.2.5033 (RStudio Inc., Boston, MA, USA) [17]. A *p* value of < 0.05 was considered statistically significant (^∗^*p* < 0.05; ^∗∗^*p* < 0.01; ^∗∗∗^*p* < 0.001).

## 3. Results

### 3.1. CCL23 Serum Levels Are Significantly Elevated in BTC Patients Compared to Healthy Controls and Patients with PSC

**Figure 1:**
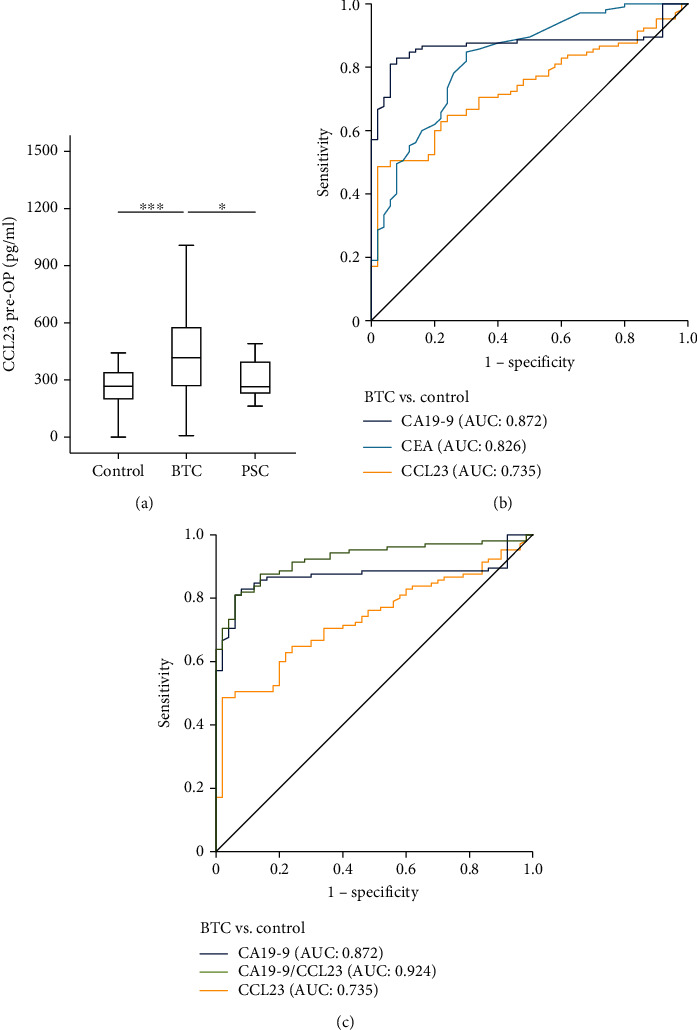
Serum levels of CCL23 are elevated in patients with BTC. (a) BTC patients show significantly elevated serum levels of CCL23 compared to healthy controls and patients with primary sclerosing cholangitis (PSC). (b) Serum CCL23 levels have a lower AUC value regarding the differentiation between BTC patients and healthy controls compared to CEA and CA19-9. (c) The combined use of CCL23 and CA19-9 increases the AUC value for the identification of BTC cancer patients.

Serum levels of CCL23 were significantly elevated in BTC patients compared to healthy controls (*p* < 0.001, Figure 1(a)). The median CCL23 level was 417.85 pg/ml compared to 266.65 pg/ml in controls. Interestingly, BTC patients also showed significantly higher CCL23 levels compared to patients with primary sclerosing cholangitis (PSC, *p* = 0.033, Figure 1(a)). In ROC curve analysis, CCL23 has an AUC of 0.735 regarding the differentiation between BTC patients and healthy controls, which was numerically higher compared to CEA (AUC_CEA_: 0.826) and CA19-9 (AUC_CA19-9_: 0.872, Figure 1(b)). At the ideal diagnostic cut-off value of 445.36 pg/ml, the diagnostic sensitivity and specificity of CCL23 was 48.6 and 98.0%, respectively. Importantly, a combined analysis of CCL23 and CA19-9 revealed a superior AUC value of 0.924 (Figure 1(c)). The diagnostic sensitivity and specificity of CCL23 and CA19-9 combined was 81.9% and 92.0%. With respect to the discrimination between BTC and PSC patients, CCL23 revealed an AUC of 0.695 (Supplementary Figure 1).

### 3.2. Analysis of CCL23 Serum Levels among Clinical Subgroups

**Figure 2:**
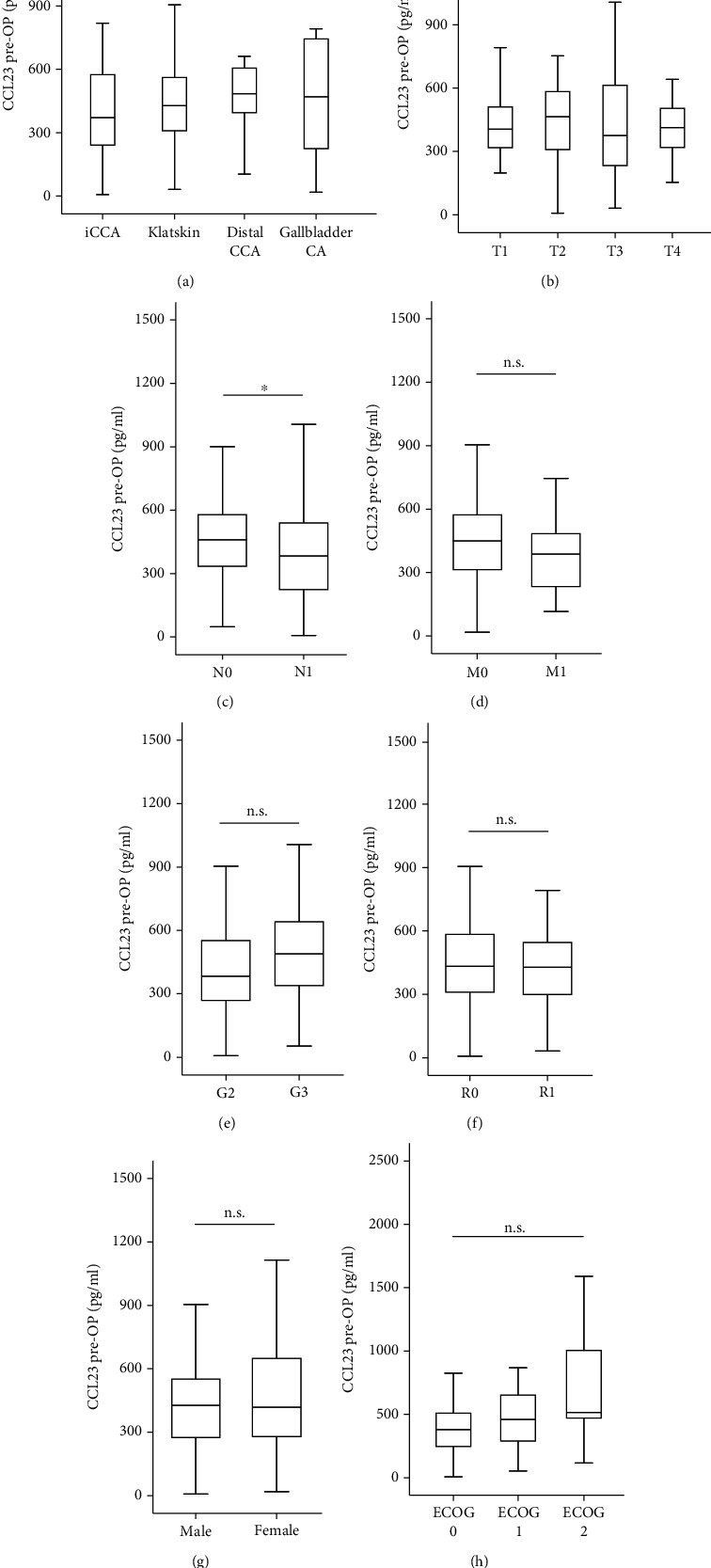
CCL23 serum levels and baseline characteristics. Baseline CCL23 levels are unaltered between BTC patients of different tumor localization (a), T-stage (b), M-stage (d), tumor grading (e), or resection status (f). (c) BTC patients with nodal positive disease (N1) have significantly lower CCL23 levels. CCL23 levels are unaltered between male and female patients (g) as well as patients with different ECOG performance status (h).

In a next step, we compared serum CCL23 levels between BTC patients of different tumor as well as clinical characteristics. Preoperative CCL23 serum levels did not differ depending on tumor localization (iCCA, Klatskin tumor, distal CCA, gallbladder cancer), T-stage, M-stage (patients who were still eligible for tumor resection), and BTC tumor grading or resection status (Figures 2(a)–2(f)). Patients with a positive lymph node status (N1), however, had significantly lower CCL23 levels (Figure 2(c)). CCL23 levels were comparable between female and male BTC patients (Figure 2(g)). Interestingly, baseline CCL23 levels stepwise increased with patients' ECOG performance status (Figure 2(h)).

To further dissect potential drivers of increased CCL23 levels among BTC patients, we performed extensive correlation analyses between baseline CCL23 levels and clinical parameters as well as various laboratory parameters. Here, we observed a positive correlation between CCL23 and patients' age (R_S_: 0.220, *p* = 0.017) and CEA serum levels (R_S_: 0.249, *p* = 0.010) as well as markers of systemic inflammation such as CRP (R_S_: 0.299, *p* = 0.001) and the leukocyte count (R_S_: 0.198, *p* = 0.032, Supplementary Table 2). In contrast, baseline CCL23 levels negatively correlated with hemoglobin levels (R_S_: -0.264, *p* = 0.004) and sodium levels (R_S_: -0.183, *p* = 0.049, Supplementary Table 2). There was neither a correlation between CCL23 and the patients' BMI not between CCL23 and liver or renal parameters (Supplementary Table 2). Finally, we observed a significant positive correlation between CCL23 and IL-4 levels (R_S_: 0.224, *p* = 0.015).

### 3.3. Elevated Preoperative CCL23 Levels Are Associated with a Poor Postoperative Survival after BTC Resection

**Figure 3:**
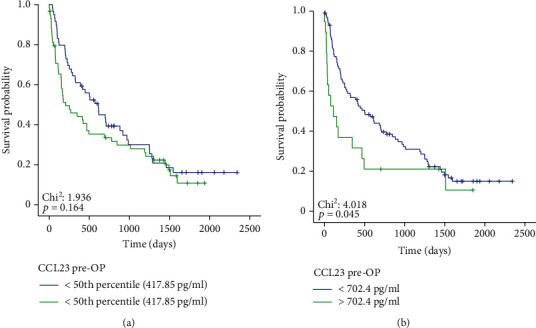
Elevated preoperative CCL23 levels are associated with an impaired outcome. (a) BTC patients with a preoperative CCL23 level above the 50^th^ percentile show a trend towards an impaired overall survival (OS) following tumor resection. (b) BTC patients with preoperative CCL23 serum levels above the optimal prognostic cut-off value (702.4.93 pg/ml) have a significantly reduced OS compared to patients with serum levels below this cut-off.

We then evaluated whether serum CCL23 levels might have a prognostic role regarding postoperative survival. Therefore, we first compared the overall survival (OS) between BTC patients with high or low (above or below the 50^th^ percentile) preoperative CCL23 levels. Kaplan-Meier curve analysis revealed that patients with CCL23 levels above the 50^th^ percentile (417.85 pg/ml) showed a nonsignificant trend towards an impaired survival compared to patients with lower CCL23 levels (Figure 3(a)). When using a calculated ideal prognostic cut-off value of 702.4 pg/ml (details on cut-off determination are shown in the Patients and Methods section), patients with high CCL23 levels above this cut-off showed a significantly impaired OS compared to patients with lower initial CCL23 serum levels (Figure 3(b)). The median OS was significantly reduced to only 110 days among patients with CCL23 levels above the ideal cut-off value compared to 501 days in patients with low CCL23 levels. The prognostic relevance of initial serum CCL23 was confirmed in Cox-regression analysis (Hazard ratio (HR): 1.001, 95% CI: 1.000-1.002, *p* = 0.016). Interestingly, the prognostic relevance of baseline CCL23 levels was only observed in female (HR: 1.001, 95% CI: 1.000-1.002, *p* = 0.008) but not male (HR: 1.001, 95% CI: 0.999-1.002, *p* = 0.457) patients and among patients < 70 years (HR: 1.001, 95% CI: 1.000-1.002, *p* = 0.027) but not among patients ≥ 70 years (HR: 1.001, 95% CI: 0.999-1.003, *p* = 0.387).

### 3.4. Postoperative CCL23 Levels and Patients' Outcome

**Figure 4:**
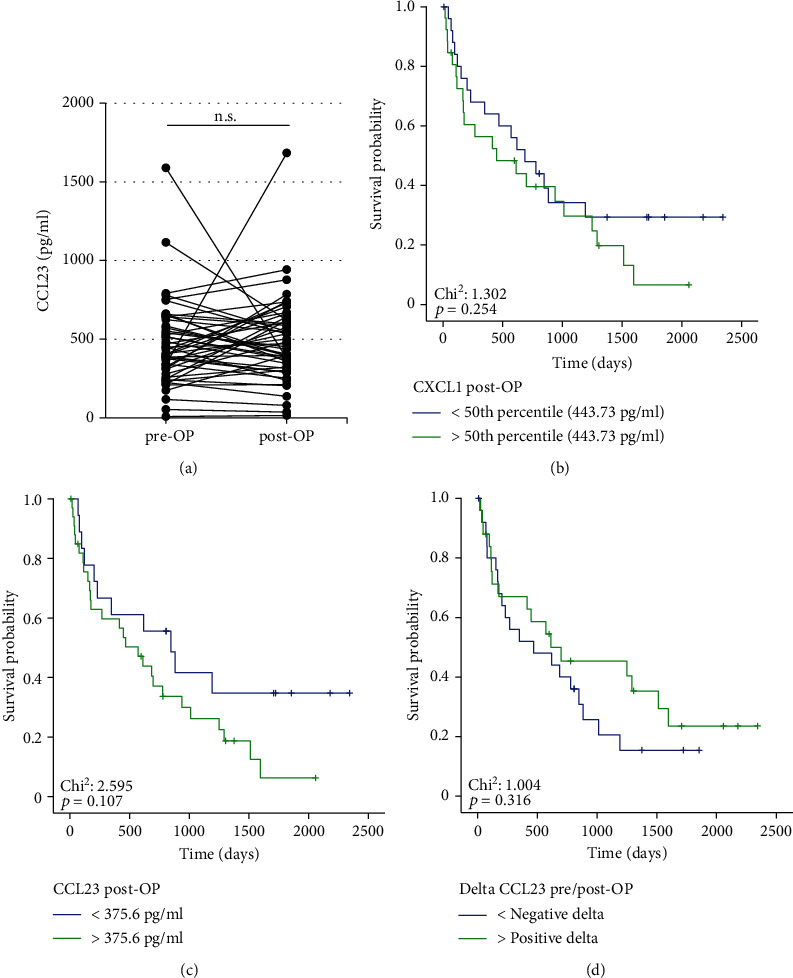
Postoperative CCL23 levels and patients' outcome. (a) Postoperative CCL23 levels do not significantly differ from the respective preoperative serum levels. (b, c) Neither the median nor an optimal prognostic cut-off value for postoperative CCL23 serum levels is able to identify BTC patients with a favorable or unfavorable postoperative outcome. (d) Individual changes of CCL23 levels before and after tumor resection (positive or negative delta) are unable to predict outcome of BTC patients.

Finally, we investigated a potential diagnostic relevance of postoperative CCL23 levels on patients' outcome. Postoperative serum samples that were taken 6-7 days after surgery were available for *n* = 51 patients and did not significantly differ with respect to the respective baseline values (Figure 4(a)). When using the 50^th^ percentile (443.73 pg/ml) as a cut-off value, we did not observe a significant difference in terms of OS between patients with high or low postoperative CCL23 levels (Figure 4(b)). Again, we established an optimal prognostic cut-off value (375.6 pg/ml) for postoperative CCL23 levels. However, although patients with a postoperative CCL23 level above the ideal cut-off value showed a trend towards an impaired survival, statistical significance was not reached (*p* = 0.107, Figure 4(c)). The individual course of CCL23 before and after surgery (increasing vs. decreasing postoperative CCL23 levels) is unsuitable to predict postoperative outcome (*p* = 0.316, Figure 4(d)). Finally, we performed correlation analyses between postoperative CCL23 levels and various laboratory parameters. However, we were unable to detect any significant correlations (Supplementary Table 3).

## 4. Discussion

At present, in most patients with early-stage BTC, the decision for or against tumor resection is primarily based on the patients' clinical performance status as well as imaging techniques providing information on the surgical resectability, while aspects of the tumor biology are not sufficiently considered [2]. In this study, we provide evidence that serum concentrations of CCL23 are elevated in patients with BTC and reflect the prognosis of BTC patients undergoing surgery since patients with initial CCL23 levels below the ideal prognostic cut-off (702.4 pg/ml) displayed a significantly longer median OS compared to the subgroup of patients with baseline CCL23 concentrations above this cut-off (501 vs. 110 days).

Similar to our data on CCL23, recently, different experimental markers were described as novel prognostic or predictive tools in cancer, however only very few markers reached clinical practice [18–20]. Similarly, the role of CCL23 in the clinical decision making for patients with BTC must be viewed with great caution. As an example, it seems unlikely and currently not reasonable that surgery on a potentially curatively resectable patient is not performed in favor of another therapeutic method, only because of an elevated CCL23 serum concentration. Nevertheless, CCL23 measurements could be integrated into existing or yet to be developed clinical decision-making tools. However, the assessment of clinical effects with medication associated toxicity in adjuvant therapy becomes even more important considering numerous trials that are currently evaluating increasingly aggressive protocols that can cause many side effects. In this context, measurements of CCL23 could help to identify patients with a prognostically unfavorable phenotype who will particularly benefit from more aggressive adjuvant therapy. Inclusion of CCL23 measurements into translational programs of these trials could provide further evidence for this hypothesis [5]. A similar development can be observed in HCC, for example, where the HAP score based on clinical and laboratory parameters is increasingly determined in the context of TACE. Importantly, in our cohort, the prognostic relevance of CCL23 was only observed in female BTC patients and patients younger than 70 years, which should lead to further investigations before a reasonable implementation into clinical decision algorithms is feasible.

Besides a well-established function in inflammatory processes and immune response, CC chemokines and their respective receptors are important elements of the pathophysiology in many different cancers. Produced by both tumor cells and tumor-associated cells such as CAF, TAM, and TAN, CC chemokines increase the proliferation, migration, and invasion of cancer cells and induce their drug resistance [21]. In ovarian cancer, macrophage-derived CCL23 was shown to contribute to an immune-suppressive tumor microenvironment by inducing an exhausted T-cell phenotype [11]. Although our study does not provide information on a functional role of CCL23 linking elevated CCL23 serum levels with an impaired outcome following resection of BTC, our data suggest that high CCL23 serum level might reflect a more aggressive cancer phenotype which is associated with a poor prognosis as seen in other tumor entities. Based on existing data on a functional induction of CCL23 by IL-4 [22], we were able to show a positive correlation between CCL23 and IL-4 serum levels. Importantly, this hypothesis clearly needs to be addressed in future molecular studies further dissecting a potential functional role of CCL23 in BTC ideally by using specific knock-out mice.

In a subgroup of patients, we analyzed levels of circulating CCL23 at the time point of one week after tumor resection to gain further insight into the longitudinal regulation of this chemokine. Interestingly, concentrations of CCL23 remained unchanged after tumor removal. This finding suggests that the tumor does not represent the major source of circulating CCL23, but that CCL23 is rather derived from the tumor microenvironment and reflect a status of systemic inflammation, which is in line with previous data [11, 23]. Concordant with this hypothesis, we observed positive correlation between baseline CCL23 concentrations and the leucocyte count as well as CRP levels, which is in good agreement with data on elevated CCL23 levels in patients with systemic inflammation [24].

We are the first to describe a potential role of CCL23 concentrations in the context of BTC. Although these data are promising, the power of our study is limited by numerous limitations that will need to be addressed in follow-up studies. Firstly, our study is only in patients from a single center, and it remains unclear whether studies in other centers would reach similar results. Secondly, only patients in early stages of disease are included, so the relevance to other stages of BTC is uncertain. In this context, our study concentrated on surgical tumor resection only and did not include alternative treatment approaches such as systemic chemotherapy. In this line of thinking, we cannot provide information regarding the question whether or not an individual patient with high baseline CCL23 levels might have benefitted to the same or even higher extend from nonsurgical therapy. Finally, our study does not allow any conclusions on a functional role of CCL23 in the context of BTC.

In summary, we provide novel evidence for a role of CCL23 levels as a previously unrecognized and easily accessible biomarker in patients with early stage BTC. If these data can be confirmed in larger, prospective, and multicenter studies, CCL23 measurements could be incorporated into future clinical decision-making algorithms for patients with BTC.

## Figures and Tables

**Table 1:** Patient characteristics.

	Patient cohort
BTC patients	*n* = 119
*Gender* [%]	
Male-female	55.1-44.9
Age [years, median, and range]	68 [37-84]
BMI [kg/m^2^, median, and range]	25.75 [18.83-46.36]
*Anatomic location of BTC* [%]	
Intrahepatic	42.0
Klatskin	40.3
Distal	10.1
Gallbladder	7.6
*Staging* [%]	
T1-T2-T3-T4	11.0-35.0-36.0-18.0
N0-N1	46.2-53.8
M0-M1	82.5-17.5
G2-G3	59.3-40.7
R0-R1	65.6-34.4
*ECOG PS* [%]	
ECOG 0	50.5
ECOG 1	40.2
ECOG 2	9.3

BTC: biliary tract cancer; BMI: body mass index; ECOG PS: “Eastern Cooperative Oncology Group” performance status.

## Data Availability

Data are available upon meaningful request from the corresponding author.

## Supplementary Materials

Supplementary Materials Supplementary Table 1: serum concentrations of CCL23 and other laboratory parameters among patients with BTC, PSC, and healthy controls. Supplementary Table 2: correlation analysis between baseline CCL23 levels and clinical as well as laboratory parameters of organ dysfunction. Supplementary Table 3: correlation analysis between postoperative CCL23 levels and laboratory parameters of organ dysfunction. Supplementary Figure 1: (A) ROC analysis for the discrimination between BTC and PSC patients.
