# Supplementary material for: Elevated Serum Levels of CCL23 Are Associated with Poor Outcome after Resection of Biliary Tract Cancer
Source: Mediators Inflamm. 2022 Dec 1;2022:6195004. doi: 10.1155/2022/6195004 (PMC9731746; doi:10.1155/2022/6195004)
Supplement: Supplementary Materials — Supplementary Table 1: serum concentrations of CCL23 and other laboratory parameters among patients with BTC, PSC, and healthy controls. Supplementary Table 2: correlation analysis between baseline CCL23 levels and clinical as well as laboratory parameters of organ dysfunction. Supplementary Table 3: correlation analysis between postoperative CCL23 levels and laboratory parameters of organ dysfunction. Supplementary Figure 1: (A) ROC analysis for the discrimination between BTC and PSC patients. [file 6195004.f1.docx]

**Elevated serum levels of CCL23 are associated with poor outcome after resection of biliary tract cancer**

**- Supplementary material -**

Christoph Roderburg^1,*^, Simon Labuhn^1,*^, Jan Bednarsch^2^, Sven A. Lang^2^, Anne T. Schneider^1^, Linda Hammerich^3^, Mihael Vucur^1^, Tom F. Ulmer^2^, Ulf P. Neumann^2^, Tom Luedde^1,#^, Sven H. Loosen^1,#^

^1^ Department of Gastroenterology, Hepatology and Infectious Diseases, University Hospital Düsseldorf, Medical Faculty of Heinrich Heine University Düsseldorf, 40225 Düsseldorf, Germany

^2^ Department of Visceral and Transplantation Surgery, University Hospital RWTH Aachen, Pauwelsstrasse 30, 52074 Aachen Germany

^3^ Department of Hepatology and Gastroenterology, Charité - Universitätsmedizin Berlin, Campus Virchow-Klinikum (CVK) and Campus Charité Mitte (CCM), Augustenburger Platz 1, 13353 Berlin, Germany

^*^ These authors share first authorship

^#^ These authors share senior authorship

## Correspondence

**Sven H. Loosen** Department of Gastroenterology, Hepatology and Infectious Diseases,

University Hospital Düsseldorf, Medical Faculty of Heinrich Heine University Düsseldorf

Moorenstraße 5, 40225 Düsseldorf, Germany

E-Mail: [Sven.Loosen@med.uni-duesseldorf.de](mailto:Sven.Loosen@med.uni-duesseldorf.de)

Phone: +49 211 81 16630

Fax: +49 211 81 04489

**Keywords:** BTC, chemokines, biomarker, BTC, cholangiocarcinoma, CCA, surgery

**Supplementary Table 1**. Serum concentrations of CCL23 and other laboratory parameters among patients with BTC, PSC and healthy controls.

| **Laboratory parameter** | **BTC patients**  (n=119) | **Healthy controls** (n=50) | **PSC**  (n=11) |
| --- | --- | --- | --- |
| CCL23 pre-OP [pg/ml] | 417.85 [7.6-2309.7] | 266.65 [32.1-652.5] | 264.87 [163.1-490.0] |
| CCL23 post-OP [pg/ml] | 443.73 [14.3-1682.9] | - |  |
| CEA [μg/l] | 2.95 [0.71-333.0] | 1.30 [0.30-6.30] |  |
| CA19-9 [U/ml] | 74.30 [0.60-38092.0] | 5.60 [0.0-44.10] |  |
| Leukocyte count [cells/nl] | 7.90 [2.90-21.60] |  |  |
| CRP [mg/l] | 17.0 [0.0-230.0] |  |  |
| Platelets [cells/nl] | 263.50 [65.0-931] |  |  |
| Hemoglobin [g/l] | 12.50 [7.80-17.10] |  |  |
| Sodium [mmol/l] | 139.50 [131.0-146.0] |  |  |
| Potassium [mmol/l] | 4.30 [2.90-6.10] |  |  |
| AST [U/l] | 45.0 [17.0-1587.0] |  |  |
| Bilirubin [mg/dl] | 0.93 [0.24-21.49] |  |  |
| ALP [U/l] | 202.0 [52.0-1055.0] |  |  |
| Creatinine [mg/dl] | 0.90 [0.42-2.1] |  |  |

BTC: biliary tract cancer, CCL23: chemokine (C-C motif) ligand 23, CEA: carcinoembryonic antigen, CA 19-9: carbohydrate-Antigen 19-9, CRP: C-reactive protein, AST: aspartate transaminase, ALP: alkaline phosphatase, PSC: primary sclerosing cholangitis

**Supplementary Table 2**. Correlation analysis between baseline CCL23 levels and clinical as well as laboratory parameters of organ dysfunction.

| **Parameter** | **CCL23** | |
| --- | --- | --- |
|  | **R_S_** | **p-value** |
| Age | **0.220*** | **0.017** |
| BMI | 0.134 | 0.150 |
| CEA | **0.249*** | **0.010** |
| CA19-9 | 0.147 | 0.134 |
| Leukocyte count | **0.198*** | **0.032** |
| CRP | **0.299*** | **0.001** |
| Platelets | 0.109 | 0.243 |
| Hemoglobin | **-0.264*** | **0.004** |
| Sodium | **-0.183*** | **0.049** |
| Potassium | -0.026 | 0.779 |
| AST | -0.001 | 0.990 |
| Bilirubin | 0.068 | 0.468 |
| ALP | 0.140 | 0.138 |
| Creatinine | 0.164 | 0.075 |
| IL-4 | **0.224*** | **0.015** |

CA19-9: carbohydrate antigen 19-9, CEA: carcinoembryonic antigen, CCL23: chemokine (C-C motif) ligand 23, CRP: C-reactive protein, AST: aspartate transaminase, ALP: alkaline phosphatase, IL: interleukin, *p<0.05

**Supplementary Table 3**. Correlation analysis between postoperative CCL23 levels and laboratory parameters of organ dysfunction.

| **Parameter**  **(post OP)** | **CCL23 post-OP** | |
| --- | --- | --- |
|  | **R_S_** | **p-value** |
| Leukocyte count | **-0.024** | **0.866** |
| CRP | **0.177** | **0.233** |
| Platelets | 0.018 | 0.903 |
| Hemoglobin | **0.040** | **0.781** |
| Sodium | **-0.134** | **0.355** |
| Potassium | 0.055 | 0.703 |
| AST | -0.230 | 0.107 |
| Bilirubin | -0.095 | 0.517 |
| ALP | -0.113 | 0.453 |
| Creatinine | 0.009 | 0.952 |

CCL23: chemokine (C-C motif) ligand 23, CRP: C-reactive protein, AST: aspartate transaminase, ALP: alkaline phosphatase.

**Supplementary Figure 1.** ROC analysis for the discrimination between BTC and PSC patients.

**
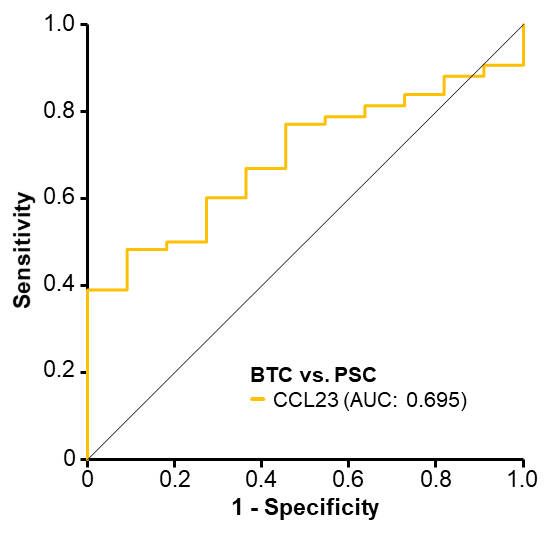
**
